# The House of Lords and the Hospitals

**Published:** 1889-05-04

**Authors:** 


### The Hospital

A Weekly Institutional Journal of

**Science, Medicine, Nursing, and Philanthropy.**

*For Hospitals, Asylums, Medical Practitioners, Students, Nurses, Families, Parishes, Congregations, and the Charitable Public.*

Vol. VI. No. 136.] [May 4, 1889.

### The House of Lords and the Hospitals.

Many years have passed by since the House of Lords has done so much really useful work as during the present Parliament. The Royal Commission on the Sweating System especially, as well as that upon Immigration, have commanded and held public attention, and great reforms may be anticipated from their labours and enquiries. It is not surprising, therefore, that steps should have been taken during the past few weeks with the view of moving the House of Lords to appoint a Commission of Enquiry into the Hospital System of the Metropolis. This is not, however, a new, but a very old question, which has exercised many active minds for upwards of ten years, as we may usefully recall to the minds of our readers.

Previous to 1883 a consensus of opinion originated a movement for an authoritative enquiry, by means of a Royal Commission, into our hospital system, as a matter of primary importance and urgency. In the previous five or six years at least four deputations, with this object, had sought interviews with the Home Secretary of the day to urge upon him the desirability of an enquiry which only a Royal Commission could satisfactorily carry through to a practical end. In this year (1883) the Hospital Conference was held, which, as the president pointed out, was entitled to be considered representative of the whole of the managers of the hospitals and infirmaries. This was so, because all had had full notice of its meetings and business, and very many had attended, so that the views of all were fairly represented. During the two days it lasted not one single word of objection to a Royal Commission was uttered by any of those who were on the committees of management, and it might therefore be taken for granted that the managing bodies of these institutions were not averse to a Royal Commission. The fact that no speaker then offered any objection to the proposal warranted the conclusion that there was no great objection, if there was, indeed, any at all, to the appointment of such a commission, which it was the object of the conference to set on foot. Despite this favourable reception, a fifth deputation arising out of the conference met with no better fate than its predecessors, and afforded one more proof that there was little to hope for from Government action or assistance. These results were arrived at because every Government has so far held that if anything were wrong in the administration of the hospitals the various independent governing bodies have the remedy in their own hands, and might introduce such changes or reforms as are needed to effect the desired improvement.

This being so, the committee appointed by the conference organised The Hospitals Association, which has steadily laboured in the direction of reform and rearrangement for the past six years. Although the association has often been abused and resisted by interested individuals, such opposition has tended to strengthen it rather than the reverse, and to day it is stronger than ever before. Its work has been quietly and unobtrusively done, but for this reason it has been of practical value, and must ultimately accomplish much, if not all, that the conference wished it to undertake in the interests of all the medical charities. It is therefore significant that the present movement in favour of a Royal Commission does not emanate from The Hospitals Association but from the Charity Organisation Society, which is acting entirely on its own initiative, and for its own purposes, apart altogether from the hospitals and medical charities. We could have wished that the Charity Organisation Society before taking action had fulfilled its original purpose, as declared last year, by obtaining full information as to (1) the maintenance of hospitals and dispensaries in foreign cities and in provincial towns, and (2) the poor-law medical work in this country and abroad, *before* presenting any petition for the appointment of a Royal Commission. To have suddenly changed their purpose, and to have taken precipitate action, is matter for regret, as it is only calculated to defeat the objects they desire to promote.

Some idea of the vastness of the subject may be gathered from the statement that at the time of the last census the population of London, in the 38 union districts of the poor-law system, amounted to 3,831,719, whilst it now exceeds four millions, and is spread over an area of 122 square miles, equal to a square of about eleven miles to the side, while the length of the streets and roads is about 1,500 miles. As Sir Rutherford Alcock once pointed out, this population has an annual mortality of upwards of 80,000, and within the area referred to there are, scattered at random, some 200 institutions for the purpose of supplying gratuitous medical relief. The approximate number of those who seek medical relief at the several hospitals and dispensaries has been ascertained to average at least one in four of the whole population, not one in two, as erroneously stated by the Charity Organisation Society in the papers they have issued. That is to say, one out of the four million residents resort to medical charities for treatment every year. It has been well pointed out that the chief evil of this state of things consists in the alarming fact that one-fourth of the lower middle and working classes are not above seeking charity when in sickness, and contribute nothing to the expenses of their maintenance. There must be many of these recipients who could afford to pay the whole, and still more who could afford to contribute a part, if the opportunity were afforded in our public hospitals, and fewer facilities given for obtaining relief gratuitously. As it is, with such facilities, temptations to pauperise themselves by being mendicants for medical relief are everywhere thrown in the way of the people who reside in London. This is the one great glaring evil of the present system, and all others sink into relative unimportance by comparison with it.

We have not space to-day to go into the many side issues, but the circumstance that the system upon which medical relief is distributed everywhere throughout London places a premium upon the pauperisation of the people, is a monstrous fact, which calls for radical reform and speedy remedy. The key to the whole position is the payment of every medical man who serves in any capacity in any of the hospitals and kindred charities. The whole of the honorary offices should be abolished, and every member of the medical staff should be remunerated for his services. If this was done, every patient would be seen by the doctor he was anxious to consult at the hospital, the outpatients would be rapidly reduced to reasonable proportions, the general practitioners of medicine would speedily come by their own again, whilst no patient would be allowed to obtain free medical relief if he could pay something for it when ill.

This fact is generally known to and recognised by the initiated, but it has not been publicly stated before because the majority of the members, and especially the senior members, of the present honorary medical staffs of our hospitals, who have rendered such magnificent and devoted service to the poor, are known to be opposed to any such radical reform. Still, the evils now crying for redress are so serious as to demand a little plain speaking, and governors and committeemen know full well that this question of the payment of all the doctors employed is the keystone to the arch of effectual reform. Why should the poorer members of the medical profession be allowed to starve slowly to death whilst the majority of the population is tending to pauperism, because the true position is withheld from public criticism and knowledge? Why, indeed? And the sooner everybody recognises the fact and acts upon it, the bet er for the poor, the profession, and the public at large.

But apart from such considerations as these, we regret to see that the Charity Organisation Society is only petitioning for an enquiry into the medical and hospital system of London. To limit the enquiry by a Royal Commission, would be to invite certain failure. A glance at the figures and tables in "The Hospital Annual," just published, will convince everybody that this must be so, for if it costs nearly twice as much to maintain a hospital bed in London as it does in the provinces, it costs almost twice as much to maintain a bed in the provinces as it does in Scotland or Ireland. Again, if any real good is to come from such a commission, it must collect information about foreign systems and methods; that in Sweden and Norway, for example, being well adapted to meet the needs of England to-day in any rearrangement of its methods for granting free medical relief. Hence, the House of Lords if approached will probably recommend the Charity Organisation Society to carry out its original intention of collecting all the necessary information in the first instance when a fair conclusion of the desirability or otherwise of appointing a Royal Commission will be better capable of demonstration. As we have said elsewhere, we are convinced that the present moment is about the worst that could be selected for the issue of such a commission, seeing that certain movements in various directions are in progress which should be allowed time to develop to completion or collapse.

There is, however, one direction in which Parliament might move at once with effect. If a short Bill were introduced on the model of the Railway Clauses Act, laying down a set of accounts which every hospital and kindred charity should be bound to adopt and to publish annually, and enacting that no one should be permitted to erect or establish a new hospital or dispensary anywhere without first obtaining a licence from the County Council or Local Government Board authorising them to do so, the voluntary hospital system would be immensely benefited and strengthened. In these two directions a speedy reform is demanded for the protection of the public, and everyone is agreed that it is desirable to have uniformity in hospital accounts, and a check imposed upon the indiscriminate multiplication of special and other so-called hospitals, which often equally mislead and defraud those who support and those who benefit by the hospital. Mr. Labouchere might usefully bring his energies to bear upon these two points, and if he would introduce a Bill, which we should be happy to supply a draft of, it might easily be passed during the present session, as everybody is heartily in favour of these much-needed and pressing reforms.

